# Bromo- and chloro-substituted flavones induce apoptosis and modulate cell death pathways in canine lymphoma and leukemia cells - a comparative *in vitro* study

**DOI:** 10.3389/fmolb.2025.1738255

**Published:** 2026-01-12

**Authors:** Anita Dudek, Ewa Dejnaka, Joanna Sulecka-Zadka, Martyna Perz, Agnieszka Krawczyk-Łebek, Edyta Kostrzewa-Susłow, Hanna Pruchnik, Aleksandra Pawlak

**Affiliations:** 1 Department of Physics and Biophysics, Faculty of Biotechnology and Food Sciences, Wrocław University of Environmental and Life Sciences, Wrocław, Poland; 2 Department of Pharmacology and Toxicology, Faculty of Veterinary Medicine, Wrocław University of Environmental and Life Sciences, Wrocław, Poland; 3 Department of Food Chemistry and Biocatalysis, Faculty of Biotechnology and Food Science, Wrocław University of Environmental and Life Sciences, Wrocław, Poland; 4 Department of Biophysics and Neuroscience, Faculty of Medicine, Wrocław Medical University, Wrocław, Poland; 5 Department of Physiology and Pharmacology, University of Georgia, Athens, GA, United States; 6 SMART Pharmacology, Precision One Health Initiative, University of Georgia, Athens, GA, United States

**Keywords:** apoptosis induction, canine cell lines, canine models, cell death mechanism, halogenated flavonoids, translational oncology models

## Abstract

**Introduction:**

Although flavonoids are natural compounds with anti-cancer potential, their clinical application is limited due to the low bioavailability. Structural modification, such as halogenation, has been identified as a strategy to enhance drug-like properties. The rationale behind this is that halogen substituents can increase lipophilicity, alter electronic distribution, and improve interactions with cellular targets. Here, we investigated the cytotoxic mechanisms of three halogenated flavones - 4′-chloroflavone (Cl-F), 6,8-dichloroflavone (DiCl-F), and 8-bromo-6-chloroflavone (BrCl-F) - in two canine B-cell models, CLB70 (leukemia) and CLBL-1 (lymphoma), chosen for their translational relevance to human hematological cancers.

**Methods:**

Cytotoxicity was assessed by MTT assay, apoptosis by annexin V/PI staining, Bcl-2 and Bcl-XL expression by Western blotting, cell cycle distribution by flow cytometry, and DNA damage by changes in H2AX phosphorylation.

**Results:**

BrCl-F demonstrated the strongest cytotoxic activity, significantly reducing metabolic activity and increasing the proportion of apoptotic cells in both cell lines. In CLB70 cells, BrCl-F treatment was accompanied by decreased expression of Bcl-2 and Bcl-XL. DiCl-F showed moderate cytotoxicity but induced a marked increase in γH2AX levels and accumulation of cells in the G2/M phase. Cl-F exhibited weaker effects and reduced cell viability primarily at higher concentrations.

**Conclusion:**

Halogenated flavones display distinct cytotoxic profiles in canine B-cell leukemia and lymphoma models, with BrCl-F showing the highest anticancer activity. These findings support further investigation of halogenated flavones as potential anticancer agents in comparative oncology.

## 1 Introduction

Flavonoids, a diverse group of naturally occurring polyphenolic compounds present in fruits, vegetables, and various plant-derived foods, have long been recognized for their diverse biological activities, among which their anti-tumor properties stand out as one of the most significant and intensely studied (Li et al., 2020; Ullah et al., 2020; Kumar and Pandey, 2013). The interest in flavonoids as potential anti-cancer agents arises not only from epidemiological and clinical evidence - suggesting that their consumption is associated with a reduced risk of carcinogenesis and can suppress the progression of established tumors - but also from extensive *in vitro* and *in vivo* investigations demonstrating their cytotoxic and proapoptotic effects in a range of human cancer cells (Hazafa et al., 2020; Fresco et al., 2006; Ramos, 2008). These compounds are known to modulate key cellular pathways associated with oxidative stress, cell cycle arrest, and the survival and proliferation of malignant cells, thereby interfering with multiple steps of tumor development and progression (Her et al., 2012; Sitarek et al., 2024; Wong et al., 2023). One of the primary challenges impeding the translation of flavonoid-based chemoprevention or chemotherapy into clinical practice is their inherently poor bioavailability. Natural flavonoids are often subject to rapid metabolism, poor absorption, and limited systemic distribution, which collectively limit their therapeutic potential (Lapasam et al., 2019; Hatanaka et al., 2010). It is evident that natural flavonoids exhibit considerable *in vitro* potency. However, the pharmacokinetic limitations associated with these compounds have prompted the investigation of structural modifications aimed at enhancing stability, cellular uptake, and biological activity (Li et al., 2022; Teng et al., 2023). A promising strategy to overcome these limitations involves the synthetic modification of the flavonoid, especially through halogenation (Hernandes et al., 2010). Halogen atoms can profoundly modulate the physicochemical and pharmacological properties of small molecules, enhancing their lipophilicity, metabolic stability, and affinity for biological targets (Jiang et al., 2016; Dias et al., 2013). Natural and synthetic halogenated compounds - including numerous antibiotics, antimetabolites (e.g., 5-fluorouracil), and anti-cancer agents (e.g., flavopiridol) - exemplify the therapeutic importance of such modifications (Kasanah and Triyanto, 2019; Sathya et al., 2020; Vodenkova et al., 2020). Notably, the semisynthetic flavonoid flavopiridol, which features a chlorine substitution on its aromatic ring, exhibits markedly improved kinase inhibition and anti-tumor efficacy, as evidenced by its clinical evaluation in leukemia patients (Senderowicz, 1999; Baghel et al., 2025). Structural studies have revealed that the presence of halogen atoms modifies the electronic distribution and binding properties of flavonoids, facilitating stronger and more selective interactions with proteins (Mendez et al., 2017).

**FIGURE 1:**
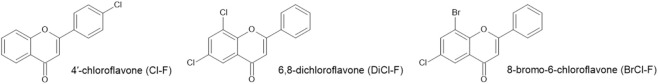
Structure of studied halogenated flavones.

In our earlier work, we demonstrated that the biological activity of halogenated flavones depends not only on the number, but also on the position of halogen atoms within the flavone structure. Using model membrane systems, including cancer-mimicking lipid bilayers, we showed that flavone with chlorine and bromine substitutions differs in its membrane-anchoring behavior, binding affinity, and impact on bilayer physicochemical properties (Dudek et al., 2024). These effects were confirmed using multiple biophysical techniques, revealing compound-specific changes in membrane fluidity, hydration, and structural organization. Moreover, we demonstrated the cytotoxic activity of halogenated flavones against the panel of canine cancer and normal cell lines with IC_50_ values ranging from about 19.44 to 74.37 µM, depending on the compound and the cell lines tested. Importantly, the same set of experiments also demonstrated that the tested halogenated flavones did not exhibit cytotoxic effects toward erythrocytes, indicating that the compounds did not adversely affect red blood cells under the tested conditions. Based on these results, we selected three halogenated flavones - 4′-chloroflavone (Cl-F), 6,8-dichloroflavone (DiCl-F), and 8-bromo-6-chloroflavone (BrCl-F) (Figure 1) - representing different halogenation patterns in terms of both halogen type and substitution position, to investigate structure–activity relationships in more detail. Given the relevance of canine models in comparative oncology - owing to their physiological and clinical similarities to humans, the spontaneous occurrence of analogous tumors, and their suitability for translational research (Pawlak et al., 2013; Pawlak et al., 2017) - studies on the effects of halogenated flavonoids in canine cancer cell lines are not only valuable but may inform both veterinary and human oncology.

The aim of the presented study was to determines the cellular mechanisms underlying the cytotoxic effects of selected halogenated flavones in the CLB70 and CLBL-1 - cell lines derived from canine B-cell leukemia and lymphoma. Specifically, we evaluated whether these compounds induce apoptotic cell death, affect the expression of anti-apoptotic proteins (Bcl-2, Bcl-XL), trigger DNA damage, and alter cell cycle progression. By integrating these analyses, we aimed to elucidate the mode of action of mono- and di-halogenated flavones in cancer cells, assess their therapeutic potential as multifunctional modulators of tumor cell fate, and identify key differences in their cellular effects. These findings may contribute to the development of flavone-based therapeutic strategies in veterinary oncology and potentially in human hematologic malignancies.

## 2 Materials and methods

### 2.1 Chemicals

The halogenated flavones investigated in this study - 4′-chloroflavone (Cl-F), 6,8-dichloroflavone (DiCl-F), and 8-bromo-6-chloroflavone (BrCl-F) - were synthesized and fully characterized in previously published works. Cl-F and DiCl-F were synthesized as described by Dudek et al. (2024) while BrCl-F was obtained according to the procedure reported by Perz et al. (2023). The purity of all halogenated flavones was confirmed by HPLC analysis, as reported in our previously published studies (Dudek et al., 2024; Perz et al., 2023). Dimethyl sulfoxide (DMSO), propidium iodide (PI), 3-[4,5-dimethylthiazol-2-yl]-2,5 diphenyl tetrazolium bromide (MTT), RIPA Lysis buffer, and SigmaFAST Protease Inhibitor Cocktail were purchased from Sigma-Aldrich (Steinheim, Germany). Annexin V-FITC and Binding Buffer 10x were purchased from Immunostep (Salamanca, Spain). Phosphate-buffered saline (PBS) was purchased from Corning Inc. (NY, USA). Water for all experiments was provided by a Milli-Q water purification system from Millipore.

### 2.2 Cell lines and cell culture

The study involved two canine cell lines: CLBL-1 (B-cell lymphoma), kindly gifted by Barbara C. Ruetgen from the Institute of Immunology, Department of Pathobiology, University of Veterinary Medicine, Vienna, Austria (Rütgen et al., 2010) and CLB70 (B-cell chronic lymphocytic leukemia) established in our laboratory (Pawlak et al., 2017). The cell lines were cultured in RPMI 1640 (Institute of Immunology and Experimental Therapy, Polish Academy of Sciences, Wrocław, Poland) or Advanced RPMI (Gibco, Grand Island, NY, USA) culture medium supplemented with 2 mM L-glutamine (Sigma Aldrich, Steinheim, Germany), 100 U/mL penicillin, 100 μg/mL streptomycin (Sigma Aldrich, Steinheim, Germany), and 10%–20% heat-inactivated fetal bovine serum (Gibco, Grand Island, NY, USA) at 37 °C in a humidified atmosphere containing 5% CO_2_.

### 2.3 Cell metabolic activity assay

Cells were seeded at a density of 2 × 10^5^ cells/mL in 96-well plates (Thermo Fisher Scientific, Roskilde, Denmark) and exposed to increasing concentrations of halogenated flavones (1.56, 3.12, 6.25, 12.5, 25, and 50 µM) for 72 h. The final concentration of DMSO in all samples did not exceed 1%, a level considered non-toxic to cells. After 72 h of incubation, 10 µL of 3-(4,5-dimethylthiazol-2-yl)-2,5-diphenyltetrazolium bromide solution (5 mg/mL) was added to each well, followed by a 4-h incubation. Subsequently, 40 µL of lysis buffer (45 g sodium dodecyl sulfate, 150 mL dimethylformamide, 183.2 mL MiliQ water) was added to each well. The optical density of the resulting formazan product was measured after 24 h using a spectrophotometric microplate reader (Spark, Tecan, Männedorf, Switzerland) at a wavelength of 570 nm. Results were obtained from at least three independent experiments (performed in triplicates) and are expressed as mean ± standard deviation (SD). IC_50_ values were calculated from dose–response curves obtained in the MTT assay using nonlinear regression.

### 2.4 Apoptosis evaluation using annexin V/PI staining

After seeding at a density of 1 × 10^5^ cells/mL in 96-well plates (Thermo Fisher Scientific, Roskilde, Denmark), the cells were incubated for 48 h with the tested compound at a concentration of 7.5, 15 and 30 µM. Following treatment, cells were collected, washed twice with PBS, and stained with annexin V-FITC and propidium iodide, in separate steps. Annexin V-FITC was added first, and cells were incubated for 10 min at room temperature in the dark. Subsequently, PI was added, followed by an additional 5-min incubation in the dark (final PI concentration 1 μg/mL). Flow cytometric analysis was immediately performed using a flow cytometer (Cytoflex, Beckman Coulter, Indianapolis, USA). Data analysis was carried out using CytExpert 2.5.0.77 software (Beckman Coulter, Indianapolis, USA).

### 2.5 Cell cycle analysis

To assess the distribution of cells across the different phases of the cell cycle, flow cytometric analysis of DNA content was performed using PI staining. Cells were cultured in 6-well plates at a density of 5 × 10^5^/mL. After 48 h of incubation with tested compounds at a final concentration of 30 μM, cells were harvested and washed twice with PBS. Cell pellets were resuspended in cold PBS and then fixed by dropwise addition of ice-cold 70% ethanol under continuous mixing to allow ethanol-induced precipitation and permeabilization. Fixed cells were stored at 4 °C for at least 24 h. Before flow cytometric analysis, ethanol was removed by centrifugation, and cells were washed with PBS. The pellets were then resuspended in a staining solution containing PI and RNase A- FxCycle™ PI/RNase Staining Solution (Thermo Fisher Scientific). Samples were incubated in the dark at room temperature for 20 min. Flow cytometric analysis was immediately performed using a flow cytometer (Cytoflex, Beckman Coulter, Indianapolis, USA). CytExpert 2.5.0.77 software (Beckman Coulter, Indianapolis, USA) was used for the data analysis.

### 2.6 Western blot analysis

A total of 5 × 10^6^ cells was rinsed with cold PBS and lysed with RIPA Lysis buffer with SigmaFAST Protease Inhibitor Cocktail and incubated for 20 min on ice. Then, after centrifuging at 10,000 rpm at 4 °C for 12 min SDS sample buffer was added to clear the supernatants and the samples were boiled at 95 °C for 5 min and subjected to SDS-polyacrylamide gel electrophoresis in a 12% gel (BioRad Mini-PROTEAN Tetra Vertical Electrophoresis Cell system, Hercules, USA). After the electrophoresis, the samples were transferred to a nitrocellulose membrane using a BioRad Mini Trans-Blot® Cell for wet transfer and then the membranes were blocked with 3% BSA in TBST at room temperature for 1 h. After blocking, the membranes were incubated overnight at 4 °C with the following murine monoclonal primary antibodies: anti-γH2A.X clone 9F3 (ab26350; Abcam, Cambridge, United Kingdom), dilution 1:1000, anti-β-actin clone C4 (sc47 778; Santa Cruz Biotechnology, California, USA), dilution 1:2000 and anti-Bcl-2 (sc-7382; Santa Cruz Biotechnology, California, USA), dilution 1:1000. One rabbit monoclonal primary antibody was also used - anti-Bcl-XL ((54H6), 2764S1; Cell Signaling Technology), dilution 1:1000. Goat Anti-Mouse Immunoglobulins/HRP (#P0447 at 1:20000 concentration in TBST solution) and Goat Anti-Rabbit Immunoglobulins/HRP (#P0448 at 1:10000 concentration in TBST solution) were used as secondary antibodies. Both secondary antibodies were from Dako (part of Agilent (United States, Santa Clara)). The membranes were incubated with the secondary antibody for 90 min at room temperature. The reaction was developed using Blotting substrate - Pierce™ ECL Western blotting Substrate (Thermo Scientific) as a substrate. Membrane visualization was performed using ChemiDoc Touch Instruments (exposure: first image, 5 s; last image, 120 s; images, 5; BioRad). For protein expression quantification, Western blot normalization with a single housekeeping protein (β-actin) was performed using Image LabTM software (version 6.1.0; BioRad).

### 2.7 Statistical analysis

All data were shown as means with standard deviation (SD). Statistical differences were analyzed using one-way ANOVA, followed by Tukey’s post hoc test, used to test statistical differences among the treatment groups and the control. Statistical analysis was performed with GraphPad Prism version 9.0.0 software (Boston, MA, USA). The results were considered significant at p < 0.05.

## 3 Results

### 3.1 Evaluation of cytotoxic activity

**FIGURE 2:**
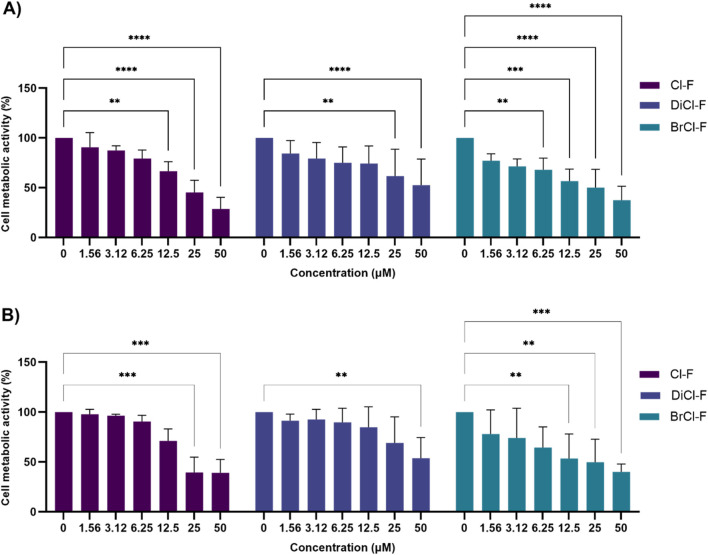
Effects of halogenated flavones - 4′-chloroflavone (Cl-F), 6,8-dichloroflavone (DiCl-F), and 8-bromo-6-chloroflavone (BrCl-F) on metabolic activity in CLBL-1 and CLB70 cell lines. Cells were treated with increasing concentrations (1.56–50 µM) of the tested compounds for 72 h, and metabolic activity was measured by the MTT assay. Data are expressed as mean ± SD from four independent experiments. Statistical significance vs. control: *p < 0.05; **p < 0.01; ***p < 0.001; ****p < 0.0001. **(A)** CLBL-1 **(B)** CLB70.

The cytotoxic effects of the halogenated flavones were assessed in CLB70 and CLBL-1 cells using the MTT assay following 72 h exposure. Cell metabolic activity was expressed as a percentage compared to untreated control (Figure 2). These data were further used to determine IC_50_ values for each compound (Table 1).

**TABLE 1:** IC_50_ values of halogenated flavones (Cl-F, DiCl-F, and BrCl-F) determined in CLB70 and CLBL-1 cell lines based on cell metabolic activity assessed by the MTT assay. IC_50_ values were calculated using nonlinear regression analysis and are presented as mean ± SD. IC_50_ values for CLB70 cells were determined in the present study, whereas IC_50_ values for CLBL-1 cells were previously reported (Dudek et al., 2024).

Cell line	Cl-F	DiCl-F	BrCl-F
IC_50_ value (µM) after 72 h of exposure to the tested compounds			
CLB70	33.88 ± 5.26	44.98 ± 17.33	24.12 ± 7.24
CLBL1 (Dudek et al., 2024)	32.75 ± 2.11	76.38 ± 23.17	38.07 ± 7.81

In CLBL-1 cells (Figure 2A), the most pronounced decrease in metabolic activity was induced by BrCl-F, which significantly reduced activity at all tested concentrations, with values dropping to approximately 40%–50% of control levels. DiCl-F showed the weakest effect, with no clear concentration-dependent trend, while Cl-F showed a significant effect only at higher concentrations, lowering metabolic activity to around 40%–60%.

In CLB70 cells (Figure 2B), overall metabolic activity remained higher compared to CLBL-1, indicating lower sensitivity to the tested treatment (Figure 2B). Similar to CLBL-1, DiCl-F displayed the weakest effect, with a significant reduction observed only at a concentration of 50 µM. BrCl-F was the most potent compound, significantly decreasing metabolic activity at a concentration of 12.5, 25, and 50 μM, to 52%, 46%, and 45%, respectively. Cl-F also lowered cell metabolic activity at 25 and 50 μM, reaching levels comparable to those observed for BrCl-F at similar concentrations. Consistent with these observations, IC_50_ analysis confirmed compound- and cell line–dependent differences in cytotoxic potency, with BrCl-F showing the highest activity in CLB70 cells and Cl-F being the most potent compound in CLBL-1 cells (Table 1).

### 3.2 Induction of apoptosis

**FIGURE 3:**
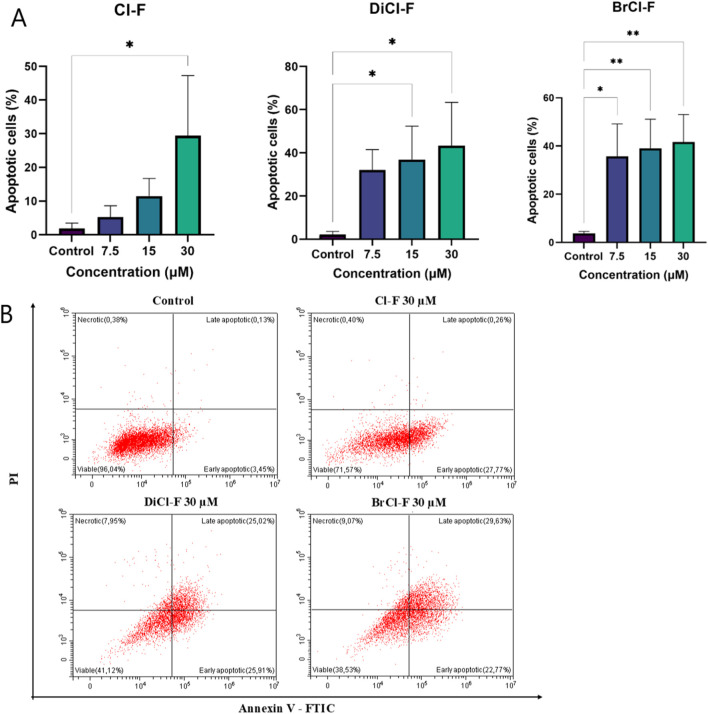
**(A)** Percentage of apoptotic CLB70 cells following 48 h exposure to Cl-F, DiCl-F, and BrCl-F at concentrations of 7.5, 15, and 30 µM. Apoptotic cells were defined as annexin V^+^ (sum of early and late apoptosis). **(B)** Representative flow cytometry dot plots showing annexin V/PI staining of CLB70 cells after 48 h treatment with Cl-F, DiCl-F, and BrCl-F. Data represent mean ± SD of three independent experiments. Statistical significance vs. control:*p < 0.05;**p < 0.01.

**FIGURE 4:**
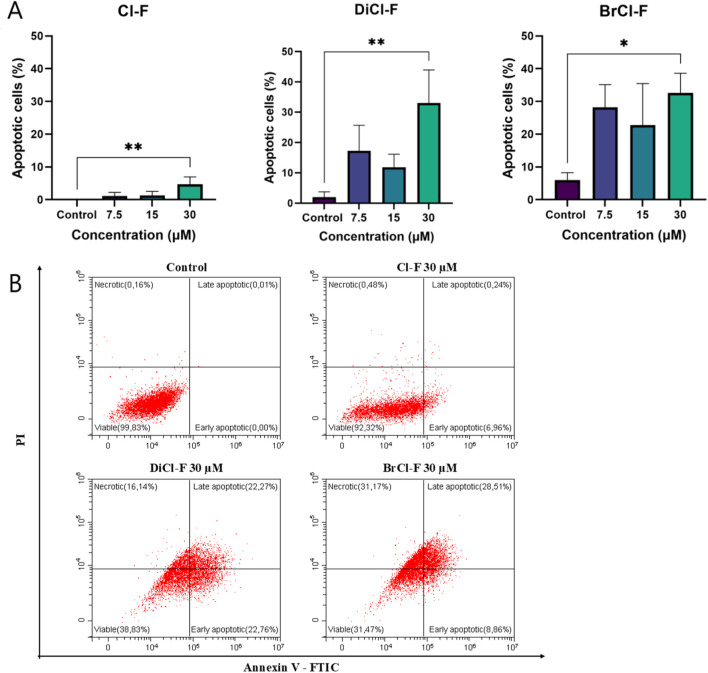
**(A)** Percentage of apoptotic CLBL-1 cells following 48 h exposure to Cl-F, DiCl-F, and BrCl-F at concentrations of 7.5, 15, and 30 µM. Apoptotic cells were defined as annexin V^+^ (sum of early and late apoptosis). **(B)** Representative flow cytometry dot plots showing annexin V/PI staining of CLBL-1 cells after 48 h treatment with Cl-F, DiCl-F, and BrCl-F. Data represent mean ± SD of three independent experiments. Statistical significance vs. control:*p < 0.05; **p < 0.01.

To assess whether the halogenated flavones induce apoptotic cell death, annexin V/PI staining followed by flow cytometry was performed after 48 h of treatment (Figures 3, 4). The percentage of apoptotic cells was calculated as the sum of early and late apoptotic populations (annexin V^+^/PI^−^ and annexin V^+^/PI^+^, respectively).

In the CLB70 cell line (Figure 3), BrCl-F induced apoptosis at all tested concentrations (7.5, 15, and 30 µM), with late apoptosis predominating over early apoptosis in the cell population. DiCl-F also increased the apoptotic fraction to 40%–50% across the tested concentrations, with early and late apoptotic cells in similar proportions. Cl-F induced apoptosis only at 30 μM, where the total apoptotic fraction reached about 30%, predominantly in the early phase, indicating slower progression toward late apoptosis compared to BrCl-F and DiCl-F.

In the CLBL-1 cell line (Figure 4), Cl-F exhibited the weakest pro-apoptotic activity, with only approximately 7% of cells undergoing early apoptosis at the highest tested concentration (30 µM) and no substantial induction of late apoptosis (Figure 4). In contrast, BrCl-F induced the highest level of apoptosis across all tested concentrations, with about 40% of the cell population undergoing programmed cell death at 30 µM. Within this apoptotic fraction, the proportion of late apoptotic cells was approximately twice that of early apoptotic cells. Treatment with DiCl-F also resulted in a notable increase in apoptosis, with late apoptotic cells predominating over early apoptotic cells at the highest concentration, although the overall apoptotic fraction remained lower than that observed for BrCl-F.

### 3.3 Expression of anti-apoptotic proteins

**FIGURE 5:**
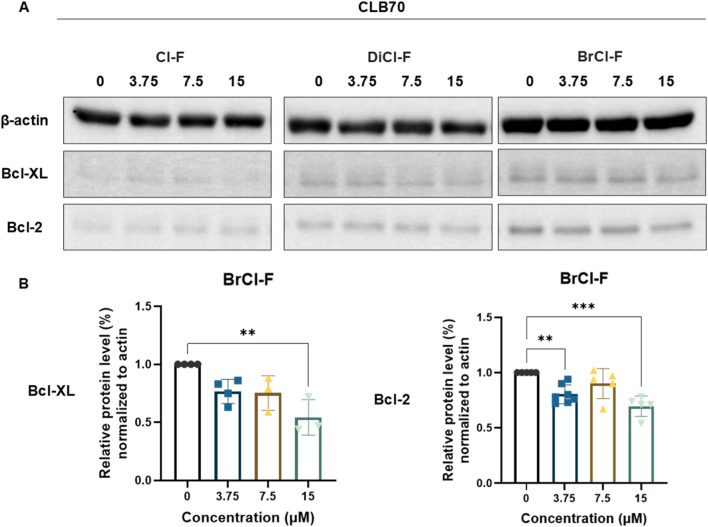
**(A)** Representative immunoblots of Bcl-XL and Bcl-2 in CLB70 cell line after 48 h treatment with Cl-F, DiCl-F, and BrCl-F at 3.75, 7.5, and 15 µm. **(B)** Relative protein expression level of Bcl-XL and Bcl-2 were normalized to β-actin protein and expressed relative to untreated cells. Data represent mean ± SD from five independent experiments. Statistical significance vs. control: *p < 0.05; **p < 0.01; ***p < 0.001.

**FIGURE 6:**
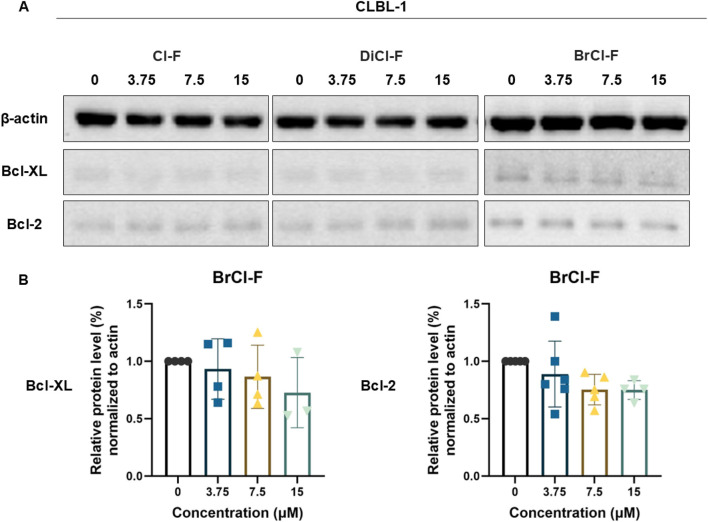
**(A)** Representative immunoblots of Bcl-XL and Bcl-2 in CLBL-1 cell line after 48 h treatment with Cl-F, DiCl-F, and BrCl-F at 3.75, 7.5, and 15 µm. **(B)** Relative protein expression level of Bcl-XL and Bcl-2 were normalized to β-actin protein and expressed relative to untreated cells. Data represent mean ± SD from five independent experiments.

To investigate whether the halogenated flavones modulate apoptotic signaling pathways at the molecular level, we evaluated the expression of two key anti-apoptotic proteins, Bcl-2 and Bcl-XL (Figures 5, 6). Cells were exposed to increasing concentrations (3.75, 7.5, and 15 µM) of Cl-F, DiCl-F, and BrCl-F for 48 h, and protein levels were assessed by Western blotting. Densitometric analysis was performed and normalized to the corresponding housekeeping protein - β-actin. Results are presented as relative expression levels compared to untreated control cells. In the CLB70 cell line (Figure 5), a significant reduction in the expression of the anti-apoptotic proteins Bcl-2 and Bcl-XL was observed exclusively following treatment with BrCl-F, with the effect becoming evident at the highest tested concentration (15 µM). A decrease in Bcl-XL expression was also apparent upon exposure to DiCl-F at 15 μM; however, this reduction did not reach statistical significance. In contrast, in the CLBL-1 cell line (Figure 6), none of the tested halogenated flavones, across the full concentration range, induced a significant alteration in the expression levels of either Bcl-2 or Bcl-XL.

### 3.4 Cell cycle analysis

**FIGURE 7:**
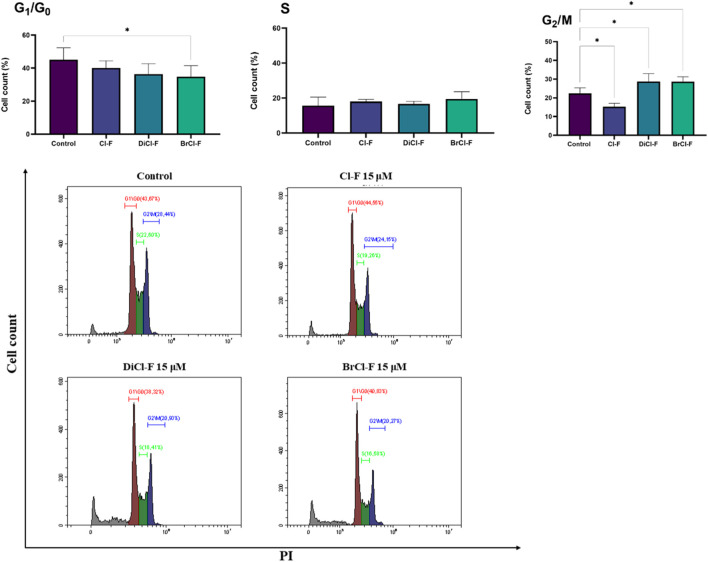
Histograms and percentage of CLB70 cells in G_0_/G_1_, S, and G_2_/M phases after exposure to 15 µM of Cl-F, DiCl-F, or BrCl-F. Data represent mean ± SD from at least four independent experiments. Statistical significance vs. untreated control: *p < 0.05.

**FIGURE 8:**
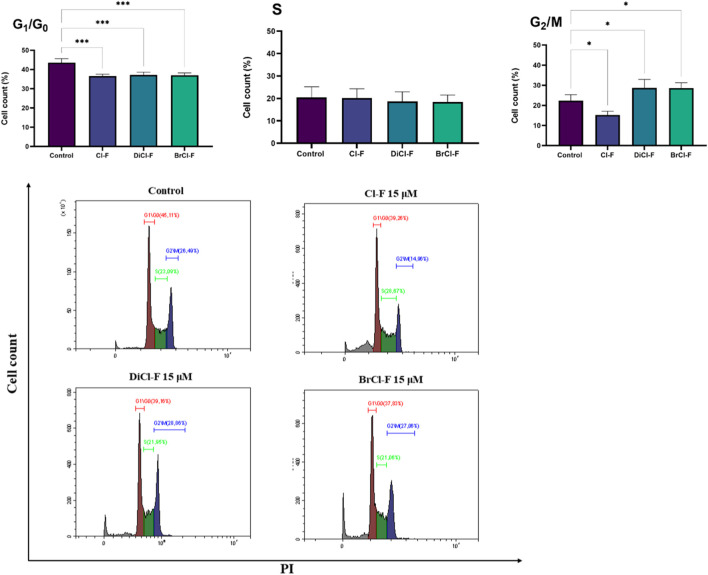
Histograms and percentage of CLBL-1 cells in G_0_/G_1_, S, and G_2_/M phases after treatment with 15 µM of Cl-F, DiCl-F, or BrCl-F. Data represent mean ± SD from three independent experiments. Statistical significance vs. untreated control: *p < 0.05.

To investigate whether the halogenated flavones affect cell cycle progression, CLBL-1 and CLB70 cells were treated with 15 µM of each compound for 48 h, followed by PI staining and flow cytometric analysis. The percentage of cells in each phase of the cell cycle (G_0_/G_1_, S, and G_2_/M) was determined and compared to untreated control (Figures 7, 8).

Cell cycle analysis of CLB70 cells (Figure 7) revealed that treatment with 15 µM of halogenated flavones for 48 h resulted in significant changes in phase distribution. The percentage of cells in the G_0_/G_1_ phase decreased for all three compounds. No significant differences were observed in the S phase, with approximately 20% of cells in this phase across all groups, including the control. In contrast, the proportion of cells in the G_2_/M phase increased significantly following treatment with DiCl-F and BrCl-F, rising from ∼20% in the control to higher levels, suggesting cell cycle arrest in this phase. Significant differences were detected in the G_2_/M phase following treatment with BrCl-F, showing an accumulation of cells in this phase, which suggests that the compound may induce G_2_/M arrest. Moreover, the growing population of dead cells visible as a peak on the right side of the histogram (sub-G_0_) indicates an increased population of these cells, especially after treatment with BrCl-F.

In the CLBL-1 halogenated flavones induced cell cycle alterations primarily in the G_0_/G_1_ and G_2_/M phases (Figure 8). A significant reduction in the G_0_/G_1_ population was observed for BrCl-F, decreasing from ∼50% in the control to below 40%. In the S phase, Cl-F treatment led to a moderate increase in the proportion of cells, while BrCl-F caused a slight decrease; however, none of these changes reached statistical significance. Similarly to the CLB70 line, a population of necrotic cells is also evident, particularly after treatment with BrCl-F.

### 3.5 Analysis of DNA damage

**FIGURE 9:**
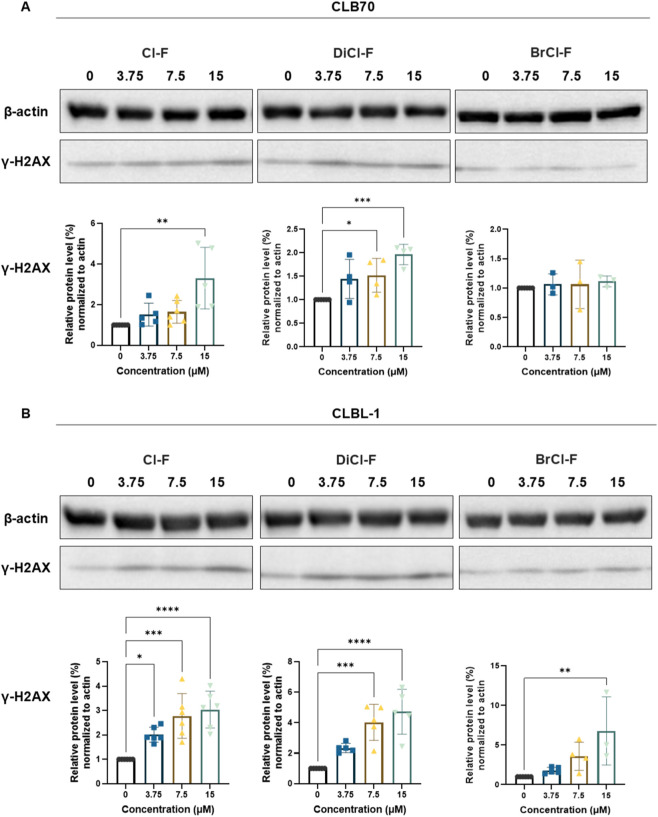
Relative expression levels and representative immunoblot of γH2AX in CLB70 **(A)** and CLBL- 1 **(B)** cells after 48 h treatment with Cl-F, DiCl-F, and BrCl-F at 3.75, 7.5, and 15 µM. Protein levels were normalized to β-actin control and expressed relative to untreated cells. Data represent mean ± SD from five independent experiments. Statistical significance vs. control: *p < 0.05; **p < 0.01; ***p < 0.001; ****p < 0.0001.

To assess whether the halogenated flavones induce DNA damage, we examined the expression levels of γH2AX, a phosphorylated form of the histone variant H2AX that accumulates rapidly at sites of DNA double-strand breaks (Mah et al., 2010). Cells were treated with 3.75, 7.5, or 15 µM of Cl-F, DiCl-F or BrCl-F for 48 h, and protein expression was analyzed by Western blotting. Densitometric quantification of γH2AX levels was performed relative to β-actin, and compared to untreated control cells (Figure 9).

In CLB70 cells (Figure 9A), a significant increase in the levels of phosphorylated H2AX (γH2AX) was observed following treatment with both Cl-F and DiCl-F. Notably, DiCl-F induced a strong and consistent upregulation at all tested concentrations, suggesting potent DNA-damaging activity. Cl-F induced a significant increase only at the highest concentration tested (15 µM). Surprisingly, BrCl-F did not cause a statistically significant elevation in γH2AX levels, despite its previously observed cytotoxic and pro-apoptotic effects in this cell line. In contrast, CLBL-1 cells (Figure 9B) exhibited a robust increase in γH2AX expression across all compounds, indicating substantial DNA damage induction. Among the tested flavones, Cl-F and BrCl-F triggered the highest levels of γH2AX accumulation, while DiCl-F showed a weaker, yet still significant, effect.

## 4 Discussion

Halogen substitution is a well-recognized strategy in medicinal chemistry to improve drug-like properties (Hernandes et al., 2010). In flavonoids, the presence of chlorine or bromine atoms modifies lipophilicity, electronic distribution, and electrophilicity, which in turn can influence membrane affinity, subcellular localization, and interactions with protein targets. Previous studies have shown that halogenation can enhance cytotoxic potential of flavonoids and related compounds in a range of cancer cell models (Saavedra et al., 2019; Gilbert et al., 2018; TOMİC et al., 2021; Marzec et al., 2020; Hurtová et al., 2022). The present study extends the observations reported in the literature to canine B-cell malignancies. Our findings demonstrate that the type of halogen and its position on the flavone framework are both critical determinants of biological activity. Our study provides a compreh ensive evaluation of the cytotoxic mechanisms of three structurally distinct halogenated flavones - 4′-chloroflavone (Cl-F), 6,8-dichloroflavone (DiCl-F), and 8-bromo-6-chloroflavone (BrCl-F) - in canine B-cell leukemia/lymphoma cell lines (CLB70 and CLBL-1). Our systematic approach, combining cytotoxicity assessment, apoptosis analysis, anti-apoptotic protein expression profiling, cell cycle evaluation, and DNA damage detection, reveals intriguing structure-activity relationships and cell line-specific responses.

It was found that BrCl-F was the most potent compound across a range of assays. This result is in accordance with the higher lipophilicity expected from the combined bromine and chlorine substitution at positions 8 and 6. The increased hydrophobic character of the compound is likely to enhance interaction with cellular membranes, facilitating uptake and perturbation of lipid bilayers. In our previous studies (Dudek et al., 2024), BrCl-F exhibited the most pronounced interaction with model cancer cell membranes among the tested halogenated flavones, leading to increased membrane’s core fluidity and lipid head reorganization. In addition, it showed the highest lipophilicity among other halogenated flavonoids. This is supported by the strong annexin V positivity observed after BrCl-F exposure, indicating phosphatidylserine externalization as an early apoptotic event. Membrane perturbation triggers apoptosis, as the reorganization of lipid asymmetry can promote mitochondrial outer membrane permeabilization and facilitate activation of the intrinsic apoptotic pathway (Vringer and Tait, 2023).

In CLB70 cells, BrCl-F further decreased Bcl-2 and Bcl-XL expression, suggesting a dual mechanism in which both direct membrane effects and interference with anti-apoptotic signaling contribute to cell death. In contrast, CLBL-1 cells exhibited robust induction of apoptosis without significant changes in Bcl-2/Bcl-XL expression, suggesting alternative mechanisms such as oxidative stress or mitochondrial depolarization. It has been reported that the synthetic chalcone 6′-benzyloxy-4-bromo-2′-hydroxychalcone which includes a bromine atom, demonstrated strong cytotoxicity against seven human leukemia cell lines with IC_50_ values in range of 2.7–8.9 μM (Saavedra et al., 2019). Moreover, halogenated chalcone was more potent than the non-halogenated or partially halogenated analogues. In the same study, it was showed that brominated chalcone induces apoptosis via activation of caspases, as well as via modulating of Bcl-2 family protein expression, decreasing anti-apoptotic Bcl-2 and increasing pro-apoptotic Bax, thereby facilitating mitochondrial-mediated apoptotic signaling. Other investigation revealed that 4′-bromoflavonol triggers apoptosis in leukemia cells, activating both intrinsic and extrinsic caspase pathways and modulating Bcl-2 family proteins (Burmistrova et al., 2014). Similar to our findings regarding CLBL-1 cell line, the authors observed that the brominated flavanol causes cell cycle arrest at the S phase, reducing the number of cells in G_1_and G_2_/M phases. However, in the CLB70 cell line we observed opposite effects, indicating the accumulation of cells and an increased proportion in the G_1_/0 and G_2_/M phases compared to the control.

The behavior of DiCl-F contrasts with that of BrCl-F. Although less effective in reducing metabolic activity, DiCl-F produced a strong and consistent increase in H2AX phosphorylation in CLB70 cells, accompanied by accumulation in the G_2_/M phase. This pattern suggests that DiCl-F predominantly induces DNA damage and activates checkpoint responses, leading to cell-cycle arrest and subsequent apoptosis. Differences in the inhibition of metabolic activity between compounds containing a chlorine atom and those with an additional bromine atom were also observed in the previously cited study (Burmistrova et al., 2014). The MTT assay demonstrated a clear advantage of the bromine-substituted flavonol over its chlorine analogue: 4′-bromoflavonol was identified as the most potent compound, with an IC_50_ value of 3.3 ± 0.7 µM, corresponding to an approximately 30-fold increase in cytotoxic activity compared with 4′-chloroflavonol, which displayed an IC_50_ of 102 ± 7 µM. The greater potency of the brominated derivative appears to result from the larger size, higher volume, and enhanced electrophilicity of the bromine atom, combined with its weaker electron-withdrawing effect at the benzene moiety relative to chlorine, collectively contributing to improved activity against cell viability. Authors also highlighted the importance of the position of the halogen atom within flavonoids framework - the position of the chlorine substituent on the B ring influenced cytotoxicity: 2′-chloroflavonol exhibited higher cytotoxicity than 4′-chloroflavonol. According to Halim et al. (2022) indicated differences in cytotoxic potency depending on the nature of the halogen substituent. Bromine substitution in the chalcone series markedly enhanced anti-proliferative potency and selectivity toward MCF-7 cells compared with the corresponding chlorinated analogues. In contrast, the pyrazoline series of the chlorinated derivatives exhibited superior activity, highlighting the structure-dependent influence of halogen substitution on cytotoxic outcomes. The type of halogen substitutes and its influence on biological activity was also pointed out in a study regarding halogenation of other aromatic compounds–coumarins. When comparing chlorinated and brominated coumarin derivatives, clear differences emerge in their cytotoxic potency and mechanisms of action. Chlorinated analogues displayed only moderate cytotoxic effects, leading to limited apoptosis and weaker disturbance of the cell cycle, with partial accumulation of cells in the G_2_/M phase. In contrast, brominated derivatives consistently showed markedly stronger activity, reducing cell viability at lower micromolar concentrations and inducing robust apoptotic responses, as evidenced by caspase activation and extensive annexin V positivity. Moreover, brominated coumarins were more effective at triggering cell cycle arrest, with a pronounced blockade at G_2_/M, thereby preventing cell proliferation. These findings suggest that the bulkier and more polarizable bromine atom enhances interactions with intracellular targets (Dettori et al., 2022).

In our study, the mono-halogenated flavone Cl-F, which contains a chlorine atom at the 3′ position of ring B, was found to consistently display the weakest effects. The activity of the compound was only evident at higher concentrations, where it reduced metabolic activity and induced early apoptosis. The limited potency of Cl-F underscores the significance of both the quantity and the configuration of substituents. Consistent with study conducted by Marzec et al. (2020), mono-halogenation at a single site, such as C-6, moderately increased cytotoxicity but was insufficient to fully potentiate apoptosis or cell cycle arrest, whereas the introduction of a second halogen atom at both C-6 and C-8 produced a synergistic effect, leading to stronger inhibition of cell proliferation, elevated ROS production, and more pronounced G_2_/M arrest. This trend was particularly evident for brominated derivatives, where the larger atomic radius, higher polarizability, and weaker electron-withdrawing character of bromine compared with chlorine appeared to facilitate more favorable interactions with hydrophobic regions of cellular targets, thereby amplifying pro-apoptotic signaling. In contrast, monochlorinated compounds, limited by smaller steric and electronic effects, lacked sufficient capacity to stabilize such interactions, which may explain their lower cytotoxic potency.

A particularly important aspect of our study is the divergence between the two cell lines. CLB70 cells, derived from a dog with chronic lymphocytic leukemia, showed higher resistance to treatment overall, requiring stronger signals such as BrCl-F-mediated Bcl-2/Bcl-XL downregulation to undergo apoptosis. DiCl-F in CLB70 mainly activated DNA-damage responses without committing cells to death, suggesting that this line possesses a higher capacity to tolerate checkpoint activation or to engage DNA repair. In contrast, CLBL-1 cells, representing a diffuse large cell lymphoma, were more sensitive to apoptosis induction, with BrCl-F triggering robust apoptosis even without changes in anti-apoptotic protein expression. This difference likely reflects intrinsic variations in baseline apoptotic priming, mitochondrial sensitivity, and DNA repair capacity between the two models. Such line-specific responses emphasize the need to test candidate compounds in multiple cellular contexts to capture the heterogeneity of tumor biology. Similar line-specific differences in drug sensitivity were also evident in our previous studies, where natural flavonoid derivatives displayed distinct activities against canine hematopoietic cancer cell lines, particularly those of leukemia and lymphoma origin (Pawlak et al., 2014; Pasaol et al., 2025; Grudzień et al., 2023).

Taken together, our findings support a model in which halogenation pattern governs the balance between apoptosis induction and DNA damage. BrCl-F preferentially engages the former, combining membrane perturbation with modulation of Bcl-2 family proteins, whereas DiCl-F primarily activates DNA-damage signaling, and Cl-F shows limited efficacy. The differences between CLB70 and CLBL-1 highlight that both the cellular background and the chemical structure of the compound are critical in shaping the response: depending on the intrinsic survival mechanisms of the target cell and the specific halogenation pattern, flavones can activate distinct molecular pathways.

## 5 Conclusion

This study demonstrates that the cytotoxic activity of halogenated flavones in canine B-cell malignancies is strongly influenced by both the type and the position of halogen substituents on the flavone structure. BrCl-F emerged as the most potent compound, combining enhanced lipophilicity with the ability to disrupt membrane integrity and modulate anti-apoptotic proteins, thereby efficiently triggering apoptosis. DiCl-F displayed a distinct profile, characterized by strong induction of DNA damage and G_2_/M arrest, while Cl-F exerted only modest effects, highlighting the limited efficacy of mono-halogenation at the 3′ position. Importantly, differences between CLB70 and CLBL-1 underline the role of cellular context, with leukemia-derived cells showing higher resistance and a greater reliance on DNA-damage signaling, and lymphoma-derived cells being more readily driven into apoptosis. Collectively, these findings emphasize that halogenation pattern and cellular background jointly dictate the balance between apoptotic and DNA-damage–related pathways, providing a basis for the rational design of flavone derivatives with optimized anti-cancer activity in both veterinary and human oncology.

Future work should aim to elucidate the kinetics and sequence of events more precisely. This should include time-resolved analyses of DNA damage, reactive oxygen species generation, mitochondrial membrane potential, and caspase activation. Comparative studies in normal canine B cells are essential for establishing selectivity. From a translational perspective, the strong apoptotic activity of BrCl-F and the DiCl-F-induced DNA damage suggest that halogenated flavones could be combined with agents that target different survival pathways, such as Bcl-2 inhibitors or DNA repair modulators. Such a strategy could enhance efficacy and potentially overcome the intrinsic resistance mechanisms present in different B-cell malignancies.

## Data Availability

The original contributions presented in the study are included in the article/supplementary material, further inquiries can be directed to the corresponding author.
